# To James Moore, Esq. Director of the National Vaccine Establishment

**Published:** 1818-01

**Authors:** 


*For the London Medical and Physical Journal.*

*To* JAMES MOORE, Esq. *Director of the National Vaccine Establishment.*

SIR,

I HAVE read your History of the Cow-pox with great satisfaction. It contains clear views of the subject, and, I doubt not, will do much good in settling the opinions of professional and non-professional men respecting Vaccination. There are, however, a few points on which I am inclined to think you have not dwelt strongly enough; while there are others to which I cannot yield my entire assent.

On these points of discrepancy I shall take the liberty of stating my sentiments without any reserve, confident that, as the whole tenor of your work proves your chief object to have been to promote the cause of truth and the success of vaccination, you will receive with candour the remarks of one who professes to be animated by motives equally honourable. As I am somewhat stinted for time, you will be pleased to overlook any want of arrangement observable in my remarks.

1st. The early period of life at which you recommend vaccination to be performed—three weeks old, has always appeared to me too soon. Independent of what you have so properly said of incompleteness of organization, infants, for a longer period than this after birth, are peculiarly liable to cutaneous affections, which may in some instances interfere with, or actually supersede, the vaccine irritation.

I am aware of its having been said that the presence of eruptions should be no impediment; on the contrary, some have even alleged that such eruptions may be benefited by the introduction of the vaccine disease into the constitution. But this, I should think, must be considered doubtful, if not dangerous, doctrine.

If the views of John Hunter with respect to the action of morbid poisons be correct, (and, in my humble judgment, they are the only views that present us with any thing like an approach to scientific precision in pathology,) then the irritation produced by the eruption already existing may so far counteract that of the vaccine virus introduced, as greatly to embarrass our judgment of the result. Even the irritation of teething, when present in a high degree, may be quite sufficient to prevent so mild a disease as the vaccine from exerting its peculiar energy; and, in fact, I have very little doubt that many of the failures have arisen from these very causes.

I respectfully suggest, therefore, that moments should be chosen for introducing the preventive when the system appears to be free from all irritation, either local or constitutional. Such moments may, under ordinary circumstances, be found. The occasion is not now so pressing as when the small-pox was stalking abroad; we can better choose our time. When the latter disease is in the neighbourhood, every consideration, as you judiciously remark, must give way.

2d. I am sorry that I feel myself compelled to dissent from the doctrine, so zealously, but (as I conceive it) so erroneously, inculcated by you, of permitting vaccination to be practised by unprofessional persons. I find the same thing countenanced by Dr. Dewar, in his "Account of an Epidemic Small-pox which occurred in Coupar, in Fife." I can perceive no satisfactory reason for this: indeed, every consideration appears to me unfavourable to it.

Unfortunately for the cause of vaccination, so insignificant was the disease held to be on its first introduction, that the most ignorant were deemed competent to superintend all its details; and the very peculiarity in its history which required and ought to have engaged the closest attention, became a reason for indifference, or justified neglect. The evils which have sprung from this source may be more easily imagined than stated.

Were the disease to render itself manifest by any outward visible signs beside those of the vesicle, or could an indication of constitutional security be accurately deduced from any thing in the state of the internal organs obvious to common observation,—then the practice of vaccination might be safely entrusted to any person of common sagacity. But, when the constitutional symptoms are of the most evanescent description; when the progress of the local affection is such as to demand the most painful attention, even of professional men, to determine accurately its genuine or spurious character; when, even at public institutions for vaccination, the difficulty of enforcing the requisite care is universally felt,—I own it does a little surprise me to find so important an office consigned to the hands of those who are so utterly unfit to perform it well. At the very time when so much stress is laid, and justly too, on the necessity of narrowly inspecting the form and progress of the vesicle,—when many of the instances of small-pox after vaccination are presumed to have arisen, either from imperfect knowledge of the true character of the vesicle, or of the cicatrix which it forms,—it were to have been wished that an injunction to commit inoculation to nurses, midwives, and other unprofessional persons, had proceeded from authority less respectable.

It is scarcely possible to avoid tracing much of the mischief which has already occurred, at least in the northern part of the kingdom (where mothers have vaccinated their children with the point of a needle, making only one puncture), in great measure to this source alone. The statement of Dr. Dewar is strongly corroborative of this opinion, whatever ingenuity and ability he may have shewn in urging the opposite doctrine: he says "it is probable a small proportion of the inoculations in Coupar were performed by medical men." If this has been the case to any extent in the other provincial towns of Scotland, there may yet be a great proportion of the vaccinated in that country in whom the liability to small-pox still exists. But, disclaiming all appearance of cavilling, and conceding to Dr. Dewar every thing he can desire, he and every one must admit that such inoculations lay the matter open to all kinds of doubt and perplexity, and that there is no dependence whatever to be placed on them in our reasonings.

People of this description are totally ignorant of those morbid phenomena in different constitutions, which may modify the action of the vaccine virus, or the appearance of the cicatrix, and regulate the opinion of the patient's security. Besides, it is among the inferior classes that small-pox is most apt to spread; and, consequently, the necessity of regular vaccination is here as great, and even greater, than among the higher ranks. The latter also can flee from the dange, if at any time they dare not encounter it; the former are chained to the spot.

I would even exclude the clergy from this duty. It is true a Treatise on the Cow-pox was published some years ago in Edinburgh,* dedicated to the Scottish clergy, and containing a strong recommendation to them to practise vaccination. The zeal of this gentleman was eminently honourable to himself, and to his profession; but I apprehend it was a misplaced zeal, nor does it appear to me to admit of a doubt that cases innumerable of spurious or imperfect

---

* By George Bell, surgeon, Edinburgh.

vaccination must have been the consequence of that proceeding. However competent to the discharge of their peculiar duties we must admit the clergy throughout the kingdom to be, it is no disparagement to them to say, that their habits and pursuits are altogether foreign to such an employment as vaccination; nor can they be expected to devote to it the necessary time and attention, even were the parents of the vaccinated to be more careful and punctual than they are generally found to be. Indeed, every interference of the kind is, in my judgment, to be seriously deprecated, and ought to be peremptorily prohibited, except in those remote situations where medical assistance is not to be had: then the clergy every-where are the best, and indeed the only, resource.

Moreover, Sir, have you not yourself declared to us that "no surgeon, however hurried with business, ought to forget, that an oversight, apparently trivial, may possibly cost his patient his sight or life?" and again, that "the observance of the instructions laid down (by you) are of more real utility than any other point of practice?" Now, Sir, I put it to your consistency, whether it be safe or judicious, where such interests as these are at stake, to confide their management to the individuals you have pointed out?

It will result, then, that the practice of vaccination must be taken out of the hands of unprofessional persons. There is no other way left but this of establishing the vaccine* on that immoveable basis on which it ought to stand. Your suggestion that the operation should be performed by all the members of the medical profession is most laudable, and ought forthwith to be adopted.

While my conviction of its paramount utility urges me thus strongly to insist on confining the practice of vaccination to our profession, I feel it would be to insult that profession were I here gravely to set about disavowing, on its behalf, all self-interested motives. Such a vindication would be spurned, as it ought to be, with contempt. Yet I cannot help observing, that, from the very first promulgation of the vaccine, a great deal of unmerited obloquy has, by the public, been cast upon that department of the profession to which I more immediately belong; and you must pardon me, Sir, for thinking that, however creditable it is to your

* By-the-bye, this word used substantively sounds rather oddly in my ear. Where is the objection to *vacciola*, seeing that we already have *variola*? But, as the term *vaccine* is probably by this time naturalised in the South, its adoption or rejection is a matter of very little consequence.

candour, you have in your late work, with reference to this point, dealt roundly enough with your brethren. It will, I suspect, be difficult to find in history a more pleasing instance of philanthropy and disinterested conduct, than that exhibited throughout the whole course of vaccination by our profession at large, and, I may venture to add, by the surgical part of it in particular. I dare safely avouch on their part that a thought of self-interest, in the sense which has been insinuated, was never for a moment suffered to harbour in their minds as a motive to influence their conduct. There are undoubtedly worthless individuals in every profession, but in the present instance I trust there are few exceptions indeed to be made; and, at all events, common charity would incline us to place the opposition which vaccination has at different times encountered from medical men to the account of misconception, ambition, rivalship, or to any other motive rather than the love of lucre, in the atrocious acceptance in which it is here understood.

3d. I observe, you very properly speak with caution of legislative interference. It certainty ought to be the last resort; yet I do not see those very formidable difficulties which have been started by many. When the vaccine was new to the world, the utmost indulgence to fears or to feelings, was necessary and even proper, for the sake of vaccination itself. But the matter now appears in a very different light. What may have been expedient and right fifteen or twenty years ago, may now be inapplicable. The great mass of the thinking part of the community, have no doubts on the subject. It is among the very lowest classes of society that the difficulties chiefly occur, and these difficulties, in most cases, do not proceed so much from ignorance or natural prejudices, as from sheer indifference and neglect.

The danger has now, in a great measure, ceased to menace, and they are consequently careless of precautions against that which is not every day staring them in the face. I never experienced any difficulty in persuading even the most obdurate of this class to have their children vaccinated when small-pox was at the door—a pretty convincing proof that their resistance had had some other foundation than conscience or feeling.

The same principle of continued non-interference carried a little farther, would go to abrogate the laws of quarantine. It is not easy, therefore, to understand why the peace and safety of the rational part of the people, who almost universally approve of vaccination, should be endangered in tenderness to the prejudices, or rather the obstinacy, of the ignorant or the bigotted, on whom neither reasoning nor experience can be expected to produce conviction.

We hear of few alarms, few ravages of small-pox in foreign countries, and, assuredly, of no small-pox after vaccination; and, I know of no better way of accounting for this, than that in all other countries the introduction of vaccination was made an affair of state concern. Persons capable of appreciating and communicating its benefits were appointed to conduct its details, and conviction of its efficacy was the invariable and immediate consequence.

It may, however, remain a question, whether the advantage may not ultimately rest with us. The probability is, that, when vaccination shall have triumphed, as it must, over all obstacles, and been established on the basis of public opinion, consolidated by long and mature discussion, it will for ever remain impregnable to attack. Though, in reply to this, it might be plausibly urged, that, were all countries rigorously to enforce vaccination, the small-pox must be speedily exterminated, and the tedious conflict of opinions and controversy consequently superseded.

4th. Another circumstance is, the period at which lymph for inoculation is directed to be taken. It is true, that, under the regulations you have laid down of procuring three or four vesicles, lymph may be taken at any period from its first formation to the acme of the disorder; but, perhaps, the common practice of waiting till the eighth day may still be adhered to with advantage. The necessity of making so many punctures offers to our notice a new view of the subject. If this be the case, what is to become of all those in whom one vesicle was thought sufficient, and they form a very great proportion of the vaccinated now existing. But, waving this matter, I suspect there can be no question that irregular vaccination must have very often occurred from the practice publicly enjoined in Scotland,* of taking lymph so early as the fifth, sixth, or seventh, day, even in those cases where only one puncture was made. I apprehend, Sir, that in every contagious disease, there must be a point in its progress at which the contagious principle, or matter, or whatever you choose to call it, is present in greater intensity than at any other. What this point is, we have no means of determining precisely. Reasoning would intimate, that the power of propagating itself must be most energetic about, or near, the moment before the disease commences its decline; and, in the disease of which I am

* Vide Treatise by Mr. George Bell.

now speaking, this would seem to be when the vesicle has assumed those peculiar appearances universally admitted to be characteristic of the disease. The formation of the areola, I presume, constitutes this period, and the eighth day is, in general, the day on which it takes place. The virus, I should suppose, ought then to be regarded as possessing its highest degree of power. I may, at least, notice what appears to be conformable to this idea, that, shortly after the introduction of vaccination, when lymph was never taken sooner than the eighth day, and only one puncture made, practitioners had the satisfaction of communicating the disorder with more certainty than they have been able to do of late. But, even admitting that the lymph does possess its highest degree of power from the moment when it first begins to appear in the cells of the vesicle, it is clear, even upon your own principles, that, whatever additional power this may give of imparting the disease to another, the chances of the vaccine exercising its protecting influence on the constitution of the individual where the lymph had been taken would be proportionally diminished, especially where only one vesicle was formed, which, for a long time, was the prevailing practice. It is impossible, therefore, to look back upon the practice at that time followed, without great fear that many failures have happened from this cause, and, still greater astonishment, that so very few have as yet shown themselves. And, really, when one reflects on the multifarious and complicated obstacles, against which vaccination has had to contend from the beginning, the wonder is not that we observe numerous cases of failure every-where starting up to alarm and perplex us, but that this extraordinary preventive has been able to maintain any thing like a respectable footing amongst us; nor can a more conclusive proof be afforded of the extent of its efficacy in destroying the susceptibility to small-pox.

All these things being duly weighed, it is matter of deep regret, when we find writers like Dr. Dewar, and the Editors of one of the London Medical Journals, express themselves doubtingly on the subject of vaccination.

I feel respect for the candour of the authors who entertain those doubts of the full efficacy of vaccination, because they are honestly and conscientiously entertained; but, in the numerous sources of failure originating in the circumstances I have pointed out, and in others that might be enumerated, ought not these respectable writers to see reason to pause before they contribute, by the influence of their authority, to shake, even in the smallest degree, the confidence of the public in this most beneficent dispensation? For what does all that they have yet witnessed amount to?—Nothing—absolutely nothing. They have allowed a few anomalous ill-authenticated cases to cast a momentary shade of doubt over their minds, and have totally lost sight of the principal link in the chain of evidence, or rather that which must be considered the grand result of all, namely, the almost complete disappearance of the small-pox.—What has charmed away this loathsome and fatal distemper?

Shall we then place one instance, in which small-pox has succeeded to vaccination, in opposition to the five hundred instances in this country, or, probably, to the five hundred thousand throughout the world, which have, for nearly twenty years, resisted that disease? This would be to adopt for our guidance the exception, and to reject the general rule.—No,—I trust we shall not so much as meditate for a moment on tamely yielding up the fortress to the enemy, on the first appearance of an insidious flag of truce; but, like brave and resolute soldiers, hold out till time and experience shall demonstrate the necessity of a surrender. If we so defend ourselves, I have few fears that we shall ever be called upon to give up the keys of the citadel.

CHIRURGUS.

*Newcastle-upon-Tyne;*
*November* 7, 1817.

---

[The author of the above has not concealed, but omitted, his name from motives of delicacy.—EDITOR.]

---

*For the London Medical and Physical Journal.*

